# Improving Risk Models for Avian Influenza: The Role of Intensive Poultry Farming and Flooded Land during the 2004 Thailand Epidemic

**DOI:** 10.1371/journal.pone.0049528

**Published:** 2012-11-19

**Authors:** Thomas P. Van Boeckel, Weerapong Thanapongtharm, Timothy Robinson, Chandrashekhar M. Biradar, Xiangming Xiao, Marius Gilbert

**Affiliations:** 1 Biological Control and Spatial Ecology, Université Libre de Bruxelles, Brussels, Belgium; 2 Fonds National de la Recherche Scientifique, Brussels, Belgium; 3 Department of Livestock Development, Bangkok, Thailand; 4 Food and Agriculture Organization of the United Nations (FAO), Rome, Italy; 5 Center for Spatial Analysis, College of Atmospheric and Geographic Sciences, University of Oklahoma, Norman, Oklahoma, United States of America; University of Liverpool, United Kingdom

## Abstract

Since 1996 when Highly Pathogenic Avian Influenza type H5N1 first emerged in southern China, numerous studies sought risk factors and produced risk maps based on environmental and anthropogenic predictors. However little attention has been paid to the link between the level of intensification of poultry production and the risk of outbreak. This study revised H5N1 risk mapping in Central and Western Thailand during the second wave of the 2004 epidemic. Production structure was quantified using a disaggregation methodology based on the number of poultry per holding. Population densities of extensively- and intensively-raised ducks and chickens were derived both at the sub-district and at the village levels. LandSat images were used to derive another previously neglected potential predictor of HPAI H5N1 risk: the proportion of water in the landscape resulting from floods. We used Monte Carlo simulation of Boosted Regression Trees models of predictor variables to characterize the risk of HPAI H5N1. Maps of mean risk and uncertainty were derived both at the sub-district and the village levels. The overall accuracy of Boosted Regression Trees models was comparable to that of logistic regression approaches. The proportion of area flooded made the highest contribution to predicting the risk of outbreak, followed by the densities of intensively-raised ducks, extensively-raised ducks and human population. Our results showed that as little as 15% of flooded land in villages is sufficient to reach the maximum level of risk associated with this variable. The spatial pattern of predicted risk is similar to previous work: areas at risk are mainly located along the flood plain of the Chao Phraya river and to the south-east of Bangkok. Using high-resolution village-level poultry census data, rather than sub-district data, the spatial accuracy of predictions was enhanced to highlight local variations in risk. Such maps provide useful information to guide intervention.

## Introduction

In January 2004 Thailand saw unprecedented epidemics of highly pathogenic avian influenza (HPAI) of the H5N1 subtype. Socio-economic impacts of the disease resulted from the losses of birds killed by the disease or by culling, and from the disruption of trade and market activities imposed by disease control measures such as movement restrictions and a temporary ban of poultry product exports [1], [2]. In addition to smallholders who raise poultry for a living and contribute significantly to home consumption, Thailand has a modern and very active commercial poultry sector, and has become one of the main exporters of poultry products in the region. The epidemics had a strong impact both on smallholders and on the commercial sector. The HPAI H5N1 virus that caused the epidemics was new to Thailand, but had been first identified in Guangdong Province of China in 1996, where it evolved before spreading internationally [3].

Thailand experienced two main epidemic waves in 2004. From January to March the first wave struck the country, and was brought under control. No outbreaks were detected from April to June but the disease returned in July. In October, following several weeks of outbreaks, Thailand decided to launch a massive survey, called the “X-ray survey” [1]. The survey involved hundreds of thousands of trained, field inspectors with the aim of producing a comprehensive view of the epidemiological situation in the field, in support of short-term responses to the epidemic, and longer-term planning of control strategies. Statistical models based on these data were used to identify risk factors, and to identify and map the main areas at risk from the disease [4], [5]. Those results helped focus surveillance and the development of control policies with regards to free-grazing ducks, the density of which had been identified as a key risk factor [5], [6]. The survey has since been repeated twice per year under the supervision of the Department of Livestock Development.

The data on epidemic wave from July 2004 to March 2005 have been analysed by several authors [4], [7]–[9]. The results depended on the risk factors considered and administrative level of analysis, but all identified domestic ducks as a key risk factor. Since 2004, HPAI H5N1 has spread to many other countries and numerous studies have analyzed HPAI H5N1 risk factors under different agro-ecological conditions in countries such as Indonesia, Bangladesh, Romania and Nigeria. Despite these numerous studies, some risk factors have been overlooked. A recent review of HPAI H5N1 spatial models [6] identified two such risk factors: flood-water and poultry production systems.

Duck farming is associated with multiple rice cropping in Thailand [10] which, in turn, implies the presence of a dense network of irrigation canals. HPAI H5N1 virus has been shown experimentally to persist in water for at least 17 days [11]. A flock with ducks infected with HPAI H5N1 could shed virus into the water of a canal and, potentially, infect a chicken farm located downstream through contaminated drinking water, even in the absence of direct contact between those two flocks (irrigation water being considered as one of the tree main sources of drinking water for poultry along with rain water and piped water when available). Water has long been suspected to play an important role in the persistence and spread of HPAI H5N1, but surprisingly few studies have included a measure of the abundance of water in the landscape as a risk factor [12], [13].

Many previous studies on HPAI H5N1 distribution have included indicators of chicken, duck or poultry abundance as a risk factor but few made an explicit distinction between the types of poultry production systems in their analyses. The link between intensification of the poultry sector and the risk of HPAI emergence and spread has received some attention [14], [15], but few studies have attempted to quantify it. One reason for this may be the distinction in poultry censuses between different types of production systems. Some studies have tried to resolve this, for example by using crude threshold values of flock sizes to define different levels of intensity in production [16] or by using anthropogenic risk factors, which are generally associated with intensive production, as surrogates [9]. Although poultry production can be categorised in many different ways, a simple approach is to separate poultry farming in two categories: extensive and intensive [15]. Otte et al. [15] define intensive production as having increased levels of inputs, of one kind or another, in order to maximize outputs; typically the yield, measured in kilograms of meat (or other product) per animal, per year. Extensive farming in this context refers to backyard production, typically with low inputs and generally used for family consumption, or sold to local markets. Poultry production in Thailand has undergone significant changes in the last decades shifting from small-scale extensive production systems towards more specialized farming involving very large flocks and increased inputs. The distinction between intensive and extensive production is important because it has implications for several epidemiological factors. These include the level of investment in animal health; bioexclusion and biocontainment measures; the absolute numbers of hosts per farm; and the potential virus load should a farm become infected. Recognising the importance of this distinction, Van Boeckel *et al* [17] developed a method to distinguish extensive from intensive chicken and duck farms based on holding size, as determined from the 2004 X-ray poultry census data in Thailand. This was used to produce detailed maps of chicken and ducks raised in extensive and intensive production systems.

This study aimed to revise some of the previous HPAI H5N1 statistical modeling of HPAI H5N1 risk in Thailand in order to test recently developed poultry production structure variables. In addition, we also tested some risk factors related to the presence of water. We also aimed to improve over previous studies carried out at the sub-district level (3^rd^ administrative level) by analyses carried out at the village level (4^th^ administrative level). Finally, we used a different modeling approach, namely Boosted Regression Trees (BRT; [18]), a method inspired by the non-parametric classification and regression trees methods that is of increasing use in epidemiology.

## Methods

### Data

**Figure 1:**
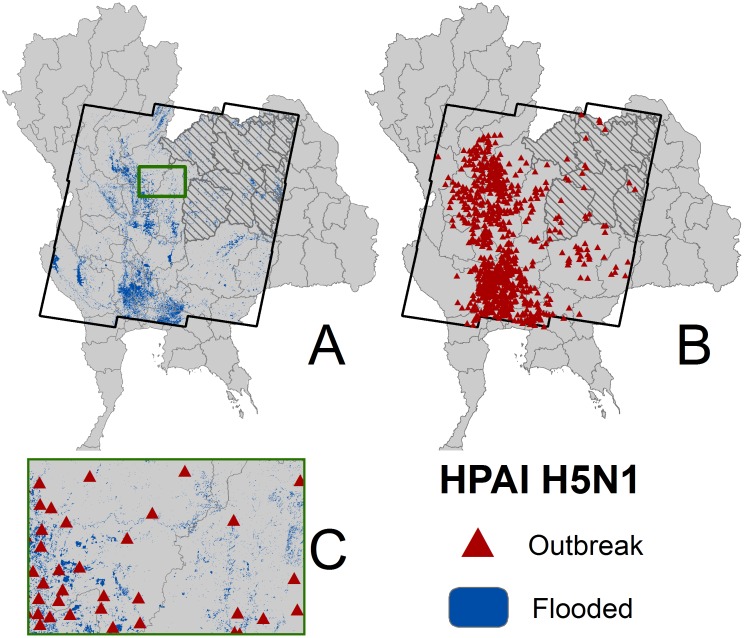
Distribution of HPAI H5N1 outbreaks and flooded areas. The distribution of flooded areas (A) and Higly Pathogenic Avian Influenza outbreaks (B) follow a comparable distribution pattern in the Central-Eastern region of Thailand and at local scale in Phitsanulok and Phetchabun provinces (C). The grey zone represents the area excluded from model training due to absence of poultry data.

The study area was restricted to the east-central region of Thailand (Fig. 1) in order to match the extent of the area where water-related risk factors had been extracted from remote-sensing data. This area includes 93% (1687 cases) of the confirmed HPAI H5N1 cases that were recorded during the second wave (1814 cases).

Four datasets were used in this study: the locations of villages, the locations of laboratory-confirmed HPAI H5N1 cases, the poultry census carried out during the first X-ray survey in October 2004, and a set of additional predictor variables. All data were projected in UTM 47N (WGS84 datum).

The Thailand village data set corresponds to point-based units and represent the fourth administrative level division of the country (1 = province; 2 = district, 3 = sub-district and 4 = village). The coordinates of 29,354 villages were obtained from the Ministry of Transport for the study area except in urban area surrounding the city of Bangkok where this sub-division of the territory is not used.

The HPAI H5N1 cases data were collected in 2004 by Department of Livestock Development through their testing of suspicious cases identified by the X-ray survey active surveillance. Positives cases were confirmed by polymerase chain reaction of samples collected from dead animals. The X-ray survey was a significant change to the surveillance approach used previously so, in order to include only data collected under similar surveillance conditions, the data considered in this study were restricted to those collected after 1 October 2004.

The poultry census data collected during the X-ray survey included counts of eight duck and chicken categories (broiler chickens, layer chickens, native chickens, broilers duck, layer ducks, Muscovy ducks, meat typed free grazing ducks and egg typed free grazing duck). These were categorized in four groups, as described in Van Boeckel *et al.* [17]: intensively raised ducks, extensively raised ducks, intensively raised chickens and extensively raised chickens. The categorization was achieved using a data-driven approach based on the number of birds per holding. The Log10-transformed numbers of birds in each category were used as predictor variables in the village level analysis, and the Log10-transformed densities of birds per km^2^ were used for the sub-district level analysis.

In addition to poultry production system variables, four other potential predictor variables were tested (Table 1, Fig. S1). As indicated by previous models [7] the number of crop cycles per year [19] and the human population density [20] were included. A third additional variable was the proportion of area covered by lakes, rivers or floods in a 1 km neighborhood around each village location. This was determined by the proportion of 30 m^2^ pixels classified as lake, river or floods in the neighborhood of each village (see below for the definition of this neighborhood). Classification of pixels as lake, river or flood was carried out by unsupervised clustering of Landsat imagery captured during the period of the second wave of the epidemic (Thanapongtharm *et al.*, submitted). In order to avoid including predictors showing co-linearity, All predictors were checked for cross correlation. All correlation coefficient values obtained were less than 0.41.

**Table 1:** Eco-climatic and anthropogenic predictors tested for correlation against presence of Highly Pathogenic Avian Influenza type H5N1.

Variable Name	Acronym	Reference	Spatial Resolution (meters)
**Mean Crops/year**	ncrop	Xiao et al 2006 [19]	500
**Presence of lake**	lake	Kmean classification clustering derived LandSat imagery	30
**Presence of River or Floods**	riverflood	Kmean classification clustering derived LandSat imagery	30
**Population Density**	ls2008	LandScan Global Population Database [50]	1000

### Preprocessing

**Figure 2:**
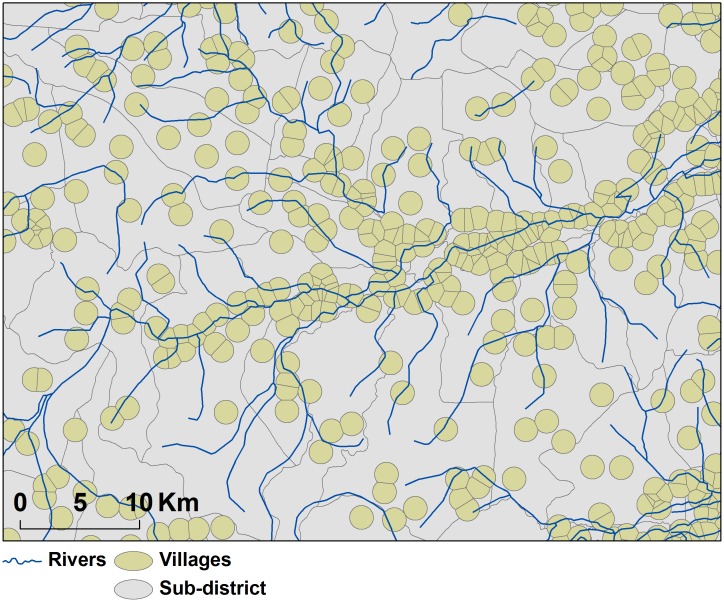
Villages “bubble” pattern in Nakhon Ratchasima Province. Villages are identified as point-based administrative units of Thailand. Their area was artificially delimited by intersecting Thiessen polygons with a one kilometre radius circular buffer.

The specific village location does not necessarily reflect the area over which animals counted in the village are distributed. In order to summarize the predictors associated with each village, it was necessary to assign each village a neighborhood area. The delineation of this neighborhood was obtained by a two-step procedure: i) Thiessen polygons were calculated around each village center in the dataset, ii) the Thiessen polygons were then intersected with a one 1 km radius buffer around each village center. This procedure restricted the neighbourhood area where villages were far apart and avoided overlap where they were close together and resulted in circular neighbourhoods in remote areas where villages were distant from each others, and in broken circles when the density of villages was high (Fig. 2). The size of the buffer was chosen according to the median minimum distance to the nearest village in our data set (0.954 km), and according to the resolution of the predictors.

**Figure 3:**
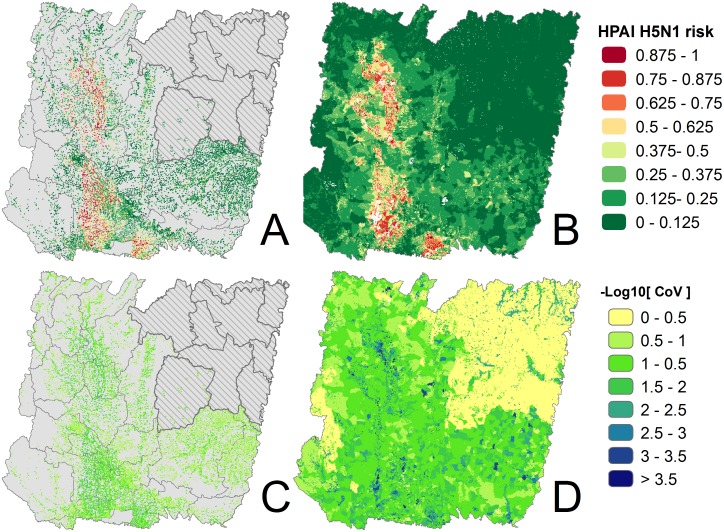
Predicted risk of HPAI outbreak. Maps of average predicted risk at the village level (A) and at the sub district level (B) for 25 iteration of the Boosted Regression Trees model. (C) and (D) represent the corresponding uncertainty maps (coefficient of variation, log scaled) associated with the risk prediction. The grey zone represents the area where poultry data were unavailable for training models.

For the village level analysis, predictor variables were averaged for each of the 29,354 village neighborhoods. For the sub-district level analysis, predictor variables were aggregated from the village level data and averaged over the area of the 2,569 sub-districts. The poultry data were then matched with the villages if a common identifier was available. This resulted in a dataset with matching records for 18,941 villages (65%) and 2,548 sub-districts (99%), after exclusion of an area in the North-Eastern region for which village-level data were lacking (Fig. 3). The geographical distribution of villages that could not be linked to any poultry data was mapped, and showed no obvious spatial pattern, nor apparent association with the distribution of the covariates.

The HPAI H5N1 cases were matched to the village and sub-district data sets. Each village and sub-district was assigned a disease status as follows: i) positives: for which an outbreak was confirmed during the 2nd epidemic wave, between 1 October 2004 and 1 March 2005 (including 740 villages and 561 sub-district) and ii) negatives: which had not been diagnosed positive for HPAI H5N1 between 23 January 2004 and 13 February 2009. The second step allowed to insure that villages and sub-districts categorized as negatives did not encountered HPAI during the second wave of the epidemic or any other previous or later epidemic episode, thereby limiting the risk of including false negative where HPAI H5N1 was present but that failed to be declared to the authorities during the second epidemic wave. The rate of matching between outbreak and poultry data was of 99% at the village level and 100% at the sub-district level.

### Modelling

Boosted Regression Trees (BRT) were used to model the probability of HPAI H5N1 presence as a function of the predictor variables. BRT were developed by [21] and are increasingly being used to predict species distributions [22], [23] and have also been used to predict disease risk, for example with HPAI H5N1 [24].

BRT implements boosting to regression trees. The procedure is iterative and implies fitting a first regression tree to the dependent variable, estimating the residuals, fitting a second tree to the residuals, update the predictions, estimate the residuals, fit a new regression tree to the new residuals, etc… The iterative loop is continued until there is no gain in predictability in adding new trees. When the response variable is binomial, the predictions of BRT are a probability of presence estimated on a scale of 0 to 1. These are estimated by adding the prediction of each regression tree multiplied by a parameter called the learning rate, that is implemented to allow a gradual fitting process. The procedure is explained in details in Elith et al. [2008] in the sections “Boosting”, p 804, and “How multiple trees produce curvilinear functions”, p 811. BRT is reported (i) to generate better predictions than do linear regression approaches [25]; (ii) to account implicitly for interactions among predictor variables; and (iii) to allow for non-monotonous relationships between the modelled response and the predictor variables [18]. These latter two points are in contrast to the logistic regression approaches previously used to model the risk of HPAI H5N1 in Thailand [7], [8].

In order to use training data with a balanced ratio of positive and negative villages, or sub-districts in the analysis, and to assess the variability of the effects of the predictor variables, a Monte Carlo procedure was implemented to produce a random sub-sample of positive and negative cases over 25 iterations. Logistic regression was indeed shown to be quite sensitive to highly unbalanced proportion of positives and negatives [26]. The training sets tested half of the HPAI H5N1 positives (370 villages or 254 sub-district) against an equal number of negatives. BRT models do not require a balanced set of positive and negatives, but since we aimed to compare the goodness of fit of BRT and logistic models, we kept the procedure identical. The BRT parameters chosen followed those used by Martin et al. [24] to model the risk of HPAI H5N1 in China: initial number of trees = 50; training rate = 0.005; tree complexity = 5, bag fraction = 0.75, and alternative parameters were tried (Table 2). The BRT approach does not provide hypothesis tests for the significance of individual variables. However, it is possible to evaluate the relative contribution (RC) of each predictor variable in a BRT model by estimating the proportion of times that a variable is selected for a splitting knot in a tree, weighted by the squared contribution of the tree towards model improvement [27]. This contribution was estimated for each of the 100 BRT models, and averaged to give an overall RC measure for each predictor variable. A particularly interesting feature of BRT is its capacity to plot the effect of each variable on the fitted value. The profile of the fitted value and each predictive variable was also averaged over the 100 runs, to show the relationship between the predictor and the predicted values.

**Table 2:** Meta-Sensitivity analysis for Boosted Regression Trees HPAI H5N1 risk model.

BRT meta parameters	Admin. Level	AUC (model)	CORL(test)	AUC (test)	AUC 95% C.I.
**lr = 0.005; bf = 0.5**	Village	0. 863	0.476	0.773	[0.743; 0.803]
**lr = 0.005; bf = 0.5**	Sub-District	0. 862	0.414	0.737	[0.705; 0.769]
**Logit**	Sub-District	0.771	0.456	0.761	[0.727; 0.794]
**lr = 0.01; bf = 0.75**	Village	0.867	0.481	0.775	[0.729; 0.819]
**lr = 0.001; bf = 0.75**	Village	0.869	0.469	0.769	[0.732; 0.805]
**lr = 0.005; bf = 0.8**	Village	0.872	0.470	0.767	[0.737; 0.803]
**lr = 0.005; bf = 0.5**	Village	0. 863	0.476	0.773	[0.743; 0.803]

lr = learning rate; bg = bag fraction; Logit = Logistic Regression Model.

In order to compare the overall accuracy of the BRT method with that of Gilbert *et al.* [7] we also used an auto logistic regression model to characterize the risk of HPAI H5N1 at the village level. The auto logistic regression was subjected to the same sampling procedure as the BRT to use a balanced ratio of positive and negative outbreak values. In order to account for the potential bias associated with spatial autocorrelation an additional index was added as predictors variable in both the logistic regression model and the BRT model. This index is an inverse distance weighed sum of the residual spatial autocorrelation (RAC) limited to a search radius of 5 Km [28]. All analysis were conducted in R (**cran**.**r**-project.org), and the BRT runs were carried out using the cross-validation functions developed by [18].

### Evaluation and prediction

The overall accuracy of the models was estimated as the average area under the receiver operator curve of the 100 iterations (AUC). The AUC was estimated both on the basis of the data set used to train the model (“model set”), and on an evaluation set of 741 villages and 508 sub-district that were not used to train the model (“test set”). A point-based prediction map was derived from the models trained at the village level and a risk map with a resolution of 1 km^2^ was produced by applying the sub-district level model to the predictor variables.

## Results

**Figure 4:**
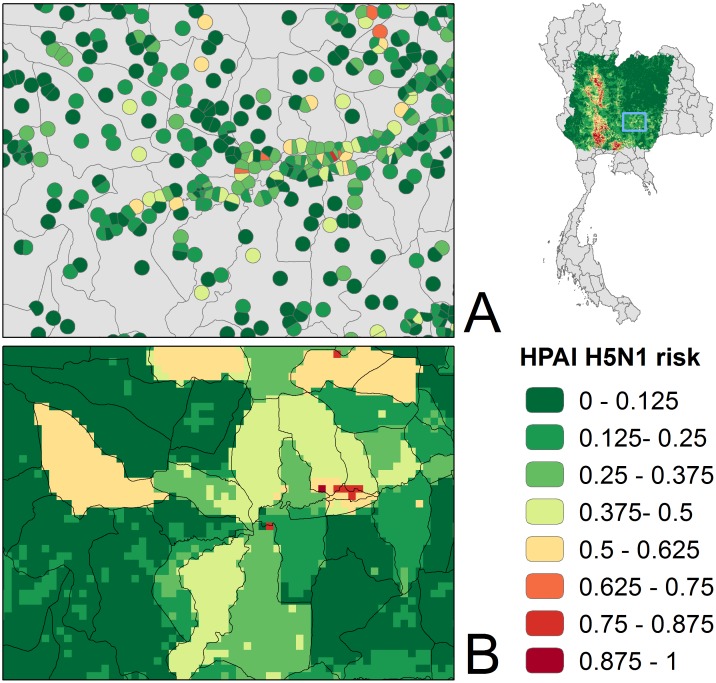
Local risk predictions (sub-district vs village). A comparison of local risk maps highlight the added value of village level prediction compared to the continuous risk surface based on sub-district poultry data. Village level poultry data allows improved targeting of potential intervention measures.

Maps of predicted HPAI H5N1 risk in the study area for the village level and at the sub district level analysis are show in Fig. 3a and Fig. 3b. The maps show a similar distribution of the risk. The uncertainty maps associated with the risk prediction are presented in Fig. 3c Fig. 3d. The median value of the coefficient of variation deviation was 18% at the sub-district level and 8% at the village level. The main areas predicted to be at risk were located along the course of the Chao Phraya river (in Suphan Buri, Nakon Sawan and Phitsanulok provinces), the Ping River (Kamphaengpet Province) and to the East of Bangkok (Chachoengchao and Nakon Nayok provinces). The risk maps provide different but complementary information: the results from the sub-district analysis display a relatively continuous description of risk across the area, due to the level of aggregation of the predictor variables, which makes no sense in areas within sub-districts where there are no people or poultry. In contrast, the village-level predictions follow the locations of the villages but do not estimate relative risk between villages. This is highlighted in Fig. 4 for an area along the Lam Takhong river in Nakon Racthasima Province.

The BRT models showed good overall agreement between observed and predicted risk of HPAI H5N1 outbreak. The mean AUC obtained for the village level and sub-district level analysis were 0.773 and 0.737 respectively; showing similar model accuracy between the village level and sub-district level models. The mean AUC obtained with a village level logistic regression model was 0.761; and were comparable to the results obtained using BRT No significant differences in mean AUC were obtained among the BRT models at the village or sub-district levels the village level logistic regression model; the mean of each falling within the 95% confidence interval of each other (Table 2). However evaluation based on the model set revealed that the BRT approach had a significantly higher degree of accuracy (AUC = 0. 862) compared with the logistic regression (AUC = 0.771). No significant change in mean AUC was observed from changing the bag fraction, within the range 0.5 to 0.8, or the learning rate, within the range 0.01 to 0.005 (Table 2), for the village level BRT model.

**Figure 5:**
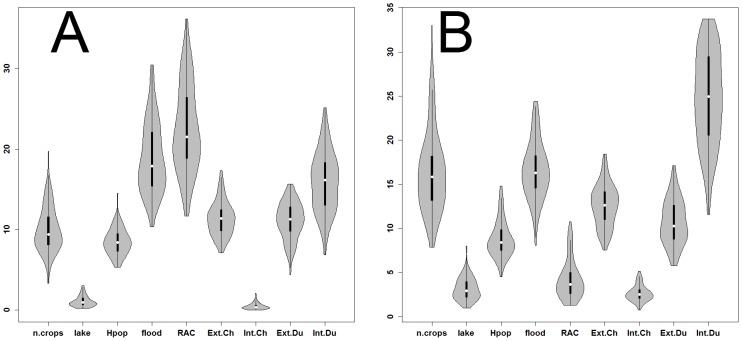
Predictors relative contribution to HPAI H5N1 risk model. Number of crop cycles (n.crops), fraction of a village neighborhood/sub-district covered with lake water (lake), human population density (Hpop, log10 scale), fraction of a village neighborhood/sub-district covered with river water or floods (floods), residual spatial autocorrelation (RAC), number/density per square kilometer of extensively raised chickens (Ext.Ch), intensively raised chickens (Int.Ch), extensively raised ducks (Ext.Du) and intensively raised ducks (Int.Du). Flooded areas and intensively raised duck shows the highest contribution to model at the village level (A) whereas at the sub-district level (B) the intensively raised ducks density is the main determinant of risk of outbreak. The relative contributions are based on the number of times a variable is selected for a node in the Boosted Regression Trees model weighted by the squared improvement to the model as a result of each node and averaged over all trees. Contributions are scaled so that the sum adds to 100.

**Figure 6:**
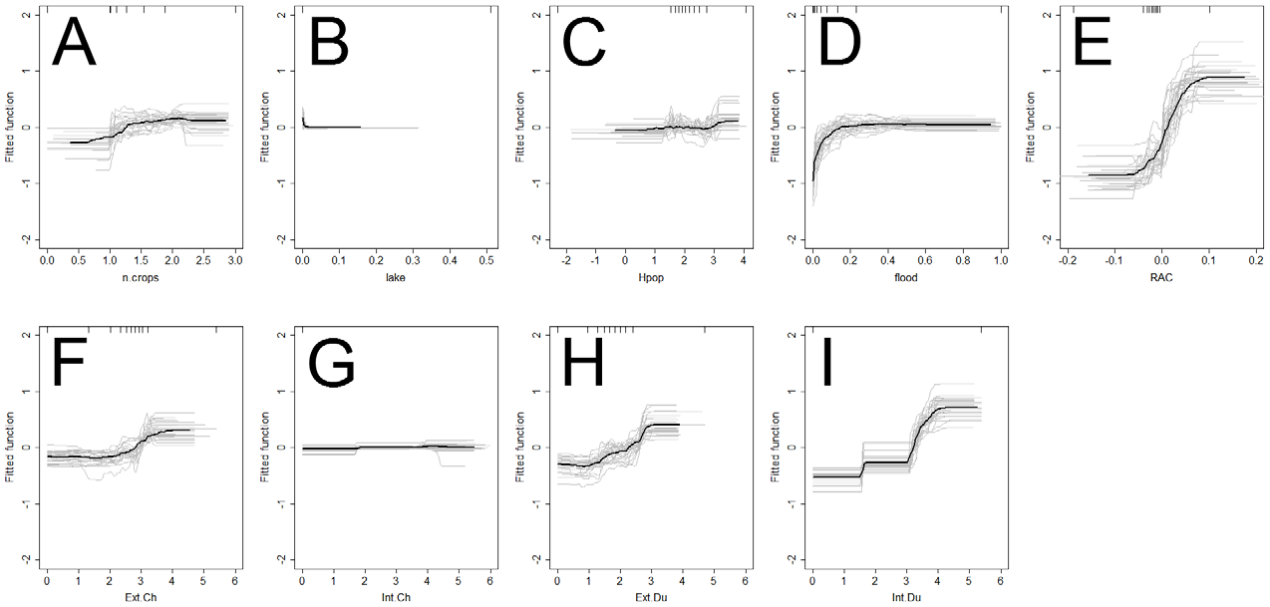
Relationship between risk factors and HPAI H5N1 fitted risk function at the village level. The HPAI H5N1 risk functions of the BRT models are plotted for the number of crop cycles (n.crops; A), fraction of a village neighborhood covered with lake water (lake; B), human population density (Hpop, log10 scale; C), fraction of a village neighborhood covered with river water or floods (floods; D), residual spatial autocorrelation (RAC, E), number of extensively raised chickens (Ext.Ch; F), intensively raised chickens (Int.Ch; G), extensively raised ducks (Ext.Du; H) and intensively raised ducks (Int.Du; I). The grey lines present the predicted line for each of the 25 iterations and the black line is the average prediction.

**Figure 7:**
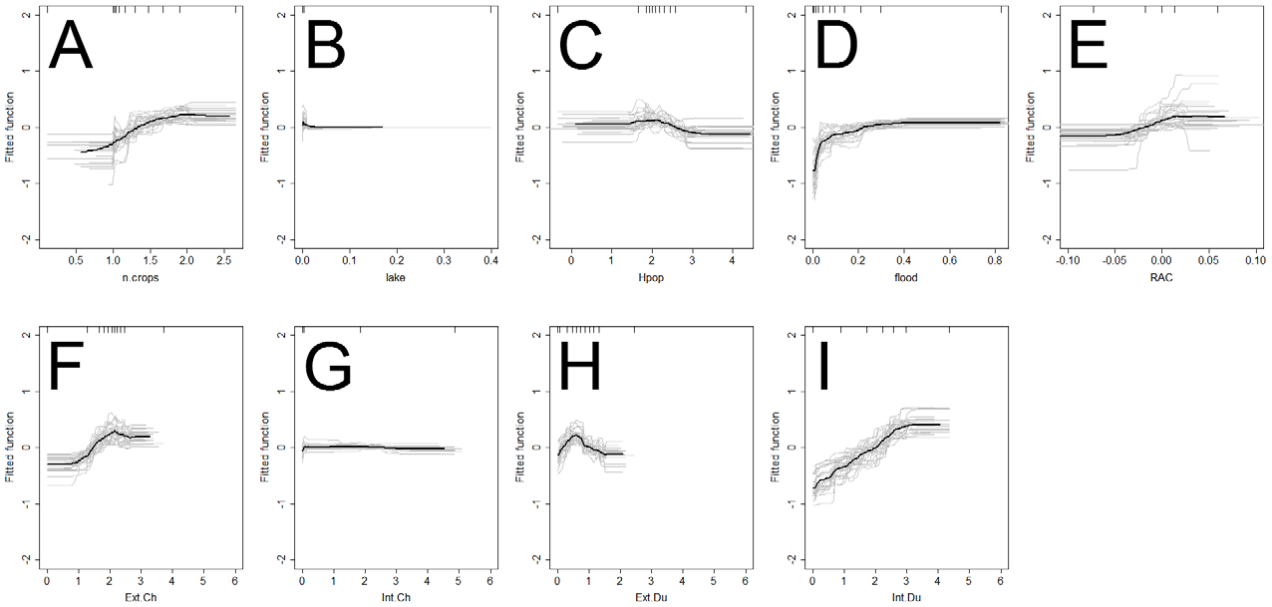
Relationship between risk factors and HPAI H5N1 fitted risk function at the sub-district level. The HPAI H5N1 risk functions of the BRT models are plotted for the number of crop cycles (n.crops; A), fraction of a village neighborhood covered with lake water (lake; B), human population density (Hpop, log10 scale; C), fraction of a village neighborhood covered with river water or floods (floods; D), residual spatial autocorrelation (RAC; E), number of extensively raised chickens (Ext.Ch; F), intensively raised chickens (Int.Ch; G), extensively raised ducks (Ext.Du; H) and intensively raised ducks (Int.Du; I). The grey lines present the predicted line for each of the 25 iterations and the black line is the average prediction.

In contrast, the relative contributions (RC) of the predictors to the BRT models (Fig. 5) and the dependency profiles between each predictor and the risk of HPAI H5N1 presence (Fig. 6 & Fig. 7) for the village level analysis were different from those of the sub-district level analysis.

The residual auto covariate predictor (RAC) had a higher RC at the village level (RC = 22.54) compared to the sub-district level (RC = 4.22), which highlights the difference of importance of the spatial autocorrelation term according the scale of the analysis, with an apparent stronger effect of spatially auto-correlated outcome at the village level than at the sub-district level.

At the village level, three groups of predictors variables could be identified based on their relative contributions to the model: important contributors, moderate and very low contributors. The proportion of area covered by rivers or floods and the number of intensively raised ducks per village formed a first group of important predictors; with RC of 17.94% and 15.93% respectively. The dependency profile for the proportion of area covered by rivers or floods Fig. 6 (d) shows that the villages with 1 to 15% of the surface covered in water have a higher risk of HPAI H5N1. Predicted risk increases with the number of ducks raised intensively up to a value of approximately 25,000 birds Fig. 5 (h). However, a low proportion of the villages hosted industrial duck farms (9.4%) and none of these fell within the lower range of bird numbers. A second group of predictor variables, which made moderate RC (8.46 to 11.36%), included the number of rice crops per year, human population and the numbers of extensively raised ducks and chickens. The dependency profile for these predictors showed that i) extensively raised birds increased the risk of HPAI H5N1 when found in high numbers, ii) the number of rice crops per year had a substantial influence on predicted risk only in areas subjected to multiple crop cycles, and iii) human population density influenced the predicted risk mostly in densely populated areas (>100 inhabitants per village). Interestingly the group of predictors with low RC (<2%) to the model included the number of chickens raised intensively and the proportion of lakes in each village. Accordingly, the dependency profiles of these two predictor variables were very flat.

At the sub-district level, one variable stood out with a high RC to the model (24.74%): the density of ducks raised intensively. Compared to the village-level analysis, the profiles of predicted risk as a function of predictor variables appeared smoother, probably resulting from the higher degree of aggregation (Fig. 6 and Fig. 7). However, the link between the predicted risk and intensively raised ducks remained prominent: the level of predicted risk increasing with increasing density of intensively raised ducks in each sub-district. Compared to the village level, a higher number of predictor variables with moderate RC were observed, ranging 8.79 to 16.60%,. These included the proportion of area covered by rivers or floods, human population density, and the density of chickens raised extensively. The proportion of area covered by rivers or floods made a lower contribution to the sub-district-level model (RC = 16.60%) compared to the village-level (RC = 17.94%) model. For chickens raised extensively, there was an increase in the predicted risk for values up to 10^2.3^ that tended to level-off, and then decrease for the highest values. The predictor variables showing the lowest contribution to the model were similar at the sub-district level and at the village level (intensively raised chickens and the proportion of lakes in each village) with the exception of the RAC (RC = 4.22).

## Discussion

The spatial distribution of HPAI H5N1 outbreaks during the second epidemic wave of 2004 has been studied by several authors [4], [7]–[9]. The analysis described in this paper builds on previous results in three areas. First, it includes some predictor variables that had not previously been evaluated: chicken numbers in extensive and intensive production systems, and variables indicative of the distribution of water in the landscape at the time of the epidemic. Second, the analysis was carried out at the village level; allowing more detailed predictions of disease risk, which may give more precise estimates of the effects of the risk factors included in the analysis. Third, an alternative statistical approach was implemented in the form of BRT; allowing the influence of each predictor variable to be evaluated across the range of its values. The emphasis was placed on the identification of risk factors and their interpretation in agro-ecological terms rather than on overall model accuracy. Accordingly, the number of predictor variables was limited to a small number of biologically meaningful variables to aid interpretation (e.g. altitude was excluded despite its predictive power as it is expected to act as a surrogate for variability within other agro-ecological factors).

As with previous studies [7]–[9], it was found that domestic ducks were an important risk factor for HPAI H5N1 in this epidemic. However, this study has further established that among those domestic ducks, the ones raised in intensive systems were more strongly associated with HPAI H5N1 presence than those raised in extensive systems. This could be explained by different factors. First, the historical practice of raising free-grazing ducks, which represents up to a third of duck production in Thailand [17], has increased in scale to acquire intensive production characteristics: flocks of several thousands of ducks are clustered in small areas with motorised transportation of birds from one rice paddy field to another [29], [30]. Given the very high numbers of ducks raised in this system, intensive logistics are required for collection and transportation of ducks and eggs, and transformation into various products [31]. These logistics may increase the risk of long-distance transmission along transportation networks. Second, in order to take advantage of economies of scale and the increased productivity associated with intensive production practices, intensively raised birds are highly selected for standard characteristics and high productivity traits: little space is left for genetic heterogeneity. Therefore flocks of genetically homogenous and intensively raised birds tends to be kept and transported in high densities. Those high densities translate into higher contact rates between individuals that favour disease transmission and facilitates perpetuation of infections at the flock level. Conversely, the number of extensively raised ducks in a village was only moderately associated with the risk of outbreaks. In many other countries, and in areas where duck production is dominated by extensive systems, duck densities have been found not to be significant risk factors, for example in Indonesia [32] and in Bangladesh [33]–[35]. It appears that within duck-producing regions, domestic ducks only contribute substantially to HPAI H5N1 risk when they are raised intensively.

The contribution of intensively raised chickens to predicted risk of HPAI H5N1 was very low, at both village and sub-district levels. This possibly results from increased bio-exclusion practices in intensive broiler and layer farms that were implemented after the first epidemic wave and have reduced the risk of farm-to-farm transmission [36], [37]. It should be noted, however, that imperfect bio-exclusion and bio-containment measures in intensive chicken farms may have largely contributed to the emergence of HPAI H5N1 in other regions [38].

It was shown that the abundance of water from rivers and floods was an important risk factor even for minor flood events. Indeed, the maximum level of predicted risk was reached if only 15% of the village neighborhood was flooded (Fig. 6 (d)), which appears to be a low threshold for regions where intensive, irrigated rice cropping is dominant (Thanapongtharm et al. Submitted). This result is consistent with the work of Thanapongtharm et al. (Submitted) based on the same dataset, but using different modeling technique, and with previous studies carried out in China [24], where a similar indicator was used for water. It is also consistent with studies in Thailand [39] and in Romania [40], where a binary variable was used to describe the presence of rivers, streams, canals or flooded land in the areas surrounding outbreaks, and was identified as a significant risk factor. The previous studies that have investigated the role of water on H5N1 outbreak locations were carried out over larger spatial extents and at coarser spatial resolutions [24]. It has been shown here that this may have influenced their results since the effect of water was stronger for the finer, village level analysis (relative contribution = 17.94%) compared to the coarser, sub-district level analysis (relative contribution = 16.60%). In the three studies mentioned above the water indicator was extracted from global or regional land use datasets. Such datasets cannot account for seasonal variability of water levels or exceptional flood events. In contrast, the water indicator that used here was produced directly from Landsat imagery collected during the second HPAI H5N1 epidemic wave (see Thanapongtharm et al. submitted) and therefore represents the actual extent of the seasonal floods at the time of the outbreaks. These results, which highlight the role of water, facilitate the interpretation of other indicators such as the number of rice crop cycles per year that have been previously identified as potential risk factors [10]. A straightforward agro-ecological interpretation was still lacking for this indicator. In this study, the fact that the presence of water from rivers and floods tends to replace the number of crop cycles in the higher resolution analysis (village level vs. sub-district level) suggests that it may better reflect the causal mechanisms that influence HPAI H5N1 risk, e.g. water-borne transmission. What these results suggest, is that the effect of cropping intensity may reflect the density of irrigated land that is required for multiple cropping, and that it is the irrigation that provides a network of streams, which in turn contributes to HPAI H5N1 spread through water contamination. Little is known about the pathways of transmission through contaminated water [41], [42]. The results presented here call for studies to be carried out based on the collection of samples from poultry and the environment, to combine information on outbreak locations, irrigation and river networks, water flow directions and sources of drinking water used in farms in order to investigate such pathways.

In contrast to the findings of [43] and [44] in China, the results presented here showed the proportion of area covered by permanent lake not to be a good predictor of HPAI H5N1 presence. The geographical context of these two studies, however, needs to be put into perspective with the density of the poultry in the landscape surrounding lakes. In China, intensive agriculture and domestic duck production is abundant in the landscape surrounding large lakes such as the Poyang Lake in Jiangxi Province. In Thailand though, such agricultural landscapes are distributed in the central plains with few permanent lakes. Another possible limit of the study is the fact that cases were detected by testing of suspicious flocks, and there is a possibility of underreporting of asymptomatic infections. Those asymptomatic infections have been reported in duck flocks, but very rarely in chicken. The study by Thanapongtharn et al. (submitted) analysed chicken and duck outbreaks separately, and found very similar risk factors and strength of associations. The fact that the same risk factors were identified in both species, suggest that asymptomatic infection that are expected to affect duck data far more than chicken data do not seem to strongly influence the identification of risk factors.

In addition to providing a finer estimate of the effect of several risk factors, the village-level model also allowed the HPAI H5N1 risk maps to be refined.. This point-based village level risk map displays somewhat differently from the continuous risk surface obtained from the sub-district level model. Both products are complementary in terms of their potential uses: A continuous risk surface has the advantage of providing an overall picture of HPAI H5N1 risk in Thailand that could be used to inform a national disease-control strategy, refine surveillance programs and, ultimately, to allocate resources to provinces with higher risk. The village-level analysis allows individual villages to be assessed in terms of risk, and lends itself to intervention organized at the village-level by veterinary officers.

In general, BRT have two main advantages over logistic regression analysis. First, they have been shown in the literature to provide a better fit to the data, even when the fit is evaluated against a separate data set, and this was already demonstrated in a comparative study [25] and more specifically for HPAI H5N1 in China [24]. BRT approaches are particularly suitable for modelling the effect of predictors that do not have a monotonic influence on risk, and are therefore better able to account for complex relationships. In this study, BRT had a much better fit than logistic regression when predictions were evaluated against the data used to train the model, but provided comparable goodness of fit when predictions were evaluated against the evaluation data set. In previous studies, such evidence of over-fitting by BRT was already noted, but the better goodness of fit was maintained when models were evaluated against a separate data set [24], which was not the case here. However, where BRT may prove more convenient to use than logistic regression approaches is in the interpretation of the results. Logistic regression models provide significance levels and regression coefficients associated with the different risk factors on the logit of the response. In contrast, BRT provides a profile of the effect of each individual predictor on the predicted outcome over the range of its values (Fig. 6). This feature enabled not only the identification of intensively raised ducks and flood events as important risk factors, but also showed the ranges of values of these predictor variables over which their effect was most important. Such information may contribute, for example, to designing a targeted surveillance strategy based on the geographical location of intensive duck production units, and seasonal flooding.

Lastly, a step forward in the way of providing evidence-based material to assist the local authorities would be to evaluate the effect of interventions strategies such as culling or vaccinations for different epidemic scenarios. However the statistical models used so far to study HPAI H5N1 are not dynamic and rely on a combination of covariates sampled at different points in time. Processes based individual models such as the ones developed by [45]–[49] are probably more suited to this purpose. However one of the major challenges of this shift of the modeling framework will lie in the ability to integrate parsimoniously the findings from statistical analyses, such as the role of intensively raised free-grazing ducks, into process based models.

## Supporting Information

Figure S1 Eastern-central region of Thailand, predictors used for Boosted Regression Trees model to predict the risk of HPAI H5N1 Outbreak.
(DOCX)
