# Supplementary figures and images for: Improving Risk Models for Avian Influenza: The Role of Intensive Poultry Farming and Flooded Land during the 2004 Thailand Epidemic
Source: PLoS One. 2012 Nov 19;7(11):e49528. doi: 10.1371/journal.pone.0049528 (PMC3501506; doi:10.1371/journal.pone.0049528)

**Figure S1**


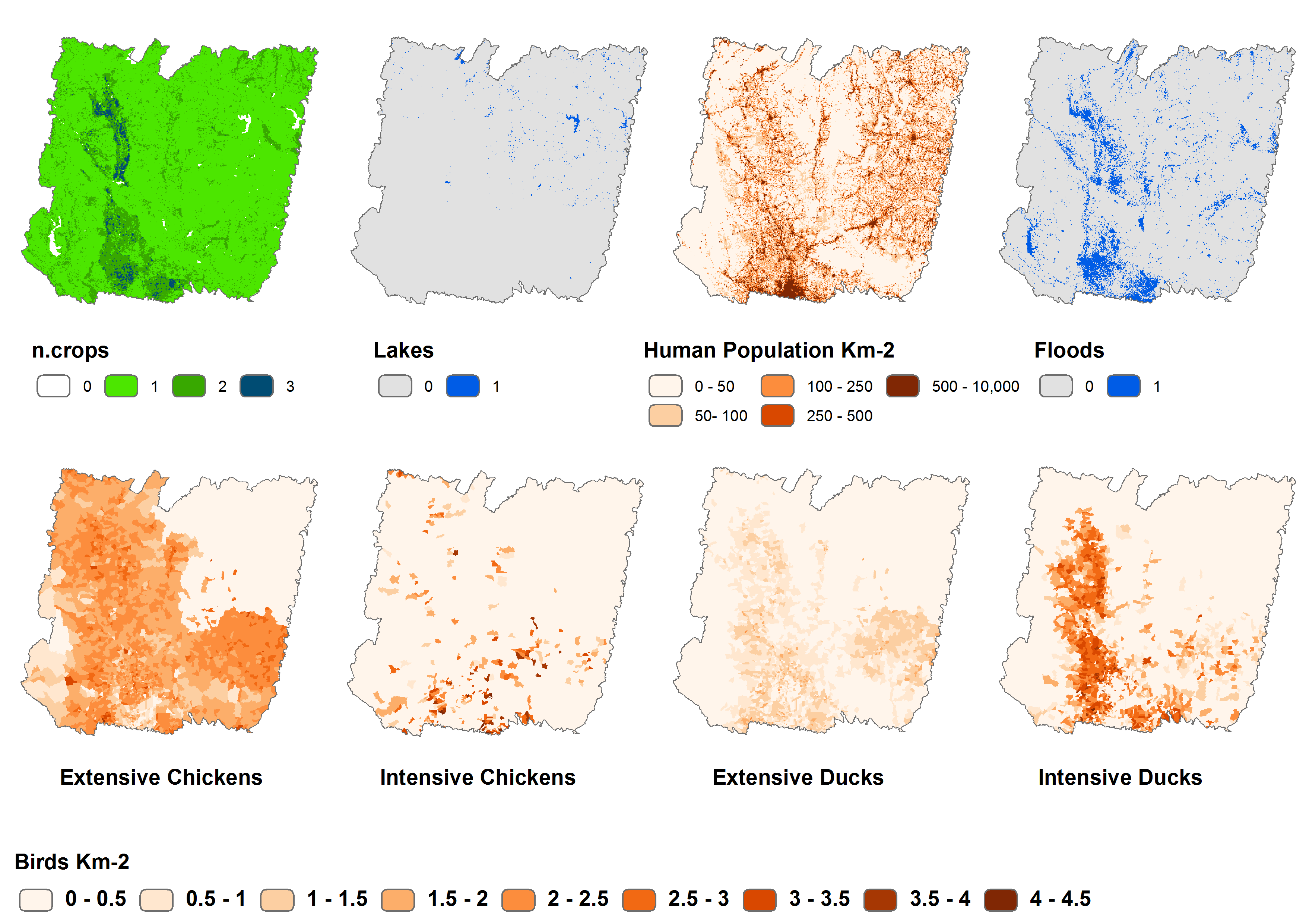

Supplement: Figure S1 — Eastern-central region of Thailand, predictors used for Boosted Regression Trees model to predict the risk of HPAI H5N1 Outbreak. (DOCX) [file pone.0049528.s001.docx]
